# High Sensitivity of Shotgun Metagenomic Sequencing in Colon Tissue Biopsy by Host DNA Depletion

**DOI:** 10.1016/j.gpb.2022.09.003

**Published:** 2022-09-26

**Authors:** Wing Yin Cheng, Wei-Xin Liu, Yanqiang Ding, Guoping Wang, Yu Shi, Eagle S.H. Chu, Sunny Wong, Joseph J.Y. Sung, Jun Yu

**Affiliations:** 1 State Key Laboratory of Digestive Disease, Institute of Digestive Disease and The Department of Medicine and Therapeutics, Li Ka Shing Institute of Health Sciences, Shenzhen Research Institute, The Chinese University of Hong Kong, Hong Kong Special Administrative Region 999077, China; 2 Lee Kong Chian School of Medicine, Nanyang Technology University, Singapore 639798, Singapore

**Keywords:** Gut microbiota, Colon tissue, Host DNA depletion, Shotgun metagenomic sequencing

## Abstract

The high host genetic background of tissue biopsies hinders the application of **shotgun metagenomic sequencing** in characterizing the tissue microbiota. We proposed an optimized method that removed host DNA from colon biopsies and examined the effect on metagenomic analysis. Human or mouse colon biopsies were divided into two groups, with one group undergoing **host DNA depletion** and the other serving as the control. Host DNA was removed through differential lysis of mammalian and bacterial cells before sequencing. The impact of host DNA depletion on microbiota was compared based on phylogenetic diversity analyses and regression analyses. Removing host DNA enhanced bacterial sequencing depth and improved species discovery, increasing bacterial reads by 2.46 ± 0.20 folds while reducing host reads by 6.80% ± 1.06%. Moreover, 2.40 times more of bacterial species were detected after host DNA depletion. This was confirmed from mouse **colon tissues**, increasing bacterial reads by 5.46 ± 0.42 folds while decreasing host reads by 10.2% ± 0.83%. Similarly, significantly more bacterial species were detected in the mouse colon tissue upon host DNA depletion (*P* < 0.001). Furthermore, an increased microbial richness was evident in the host DNA-depleted samples compared with non-depleted controls in human colon biopsies and mouse colon tissues (*P* < 0.001). Our optimized method of host DNA depletion improves the sensitivity of shotgun metagenomic sequencing in bacteria detection in the biopsy, which may yield a more accurate taxonomic profile of the tissue microbiota and identify bacteria that are important for disease initiation or progression.

## Introduction

Culture-independent assessment of the bacterial taxonomic profiles by next-generation sequencing has become a common approach to examining the relationship between dysbiosis and various diseases. Characterization of the microbial profile associated with different anatomical locations has been reported by studies that analyzed human oral, gut mucosal, and fecal samples using 16S rRNA gene amplicon or shotgun metagenomic sequencing [1]. The 16S rRNA gene sequencing is limited for its resolution of taxonomic identification, whereas metagenomic sequencing can identify bacteria at species or even strain levels [2], [3], [4]. Moreover, metagenomic sequencing can provide multi-kingdom profiling, with the implication of inferring the interplay across multiple domains [5].

The comprehensive analysis of microbial and host genetic material in samples from patients has important clinical implications, including diagnosis and treatment of disease. We previously identified the microbial marker associating colorectal carcinogenesis [6] or gastric carcinogenesis [7] using 16S rRNA gene sequencing of tissue biopsies. Metagenomic sequencing provides not only the taxonomic composition of the microbiota in the tissue, but also their potential biological functions [8]. However, the massive difference in the amount of human and bacterial DNA poses a constraint on the analysis of tissue samples by metagenomic sequencing. Low-abundance bacteria remain undetectable due to the overwhelming amount of human DNA in the tissue. Typically, greater than 99% sequencing reads from tissue samples are of human origin with less than 1% reads of non-human origin. The increased host background in tissues reduces the number and proportion of microbial reads, decreasing the metagenomic sequencing sensitivity in detecting microorganisms [9], [10]. To mitigate the concern that the majority of the sequencing reads are derived from host DNA dominant in tissue samples, measures such as targeted sequencing [11], increased sequencing depth [12], [13], or removal of host DNA prior to sequencing [14], [15], [16], [17] have been proposed. Indeed, various protocols have been reported to deplete host DNA in sputum [8], [18], saliva [19], and diabetic foot infection [20] samples, thereby increasing microbial sequencing depth and the number of detected taxa. The effects of host DNA depletion on the metagenomic analysis and microbial profiles in tissue samples remain to be elucidated. In this study, we evaluated the efficacy of metagenomic sequencing on microbiome at the species level of tissue biopsies by host DNA depletion.

## Results

### Removal of host DNA from tissue sample increased the number of microbial reads

Human and mouse colon tissues were homogenized and divided into two portions equally, with one portion undergoing host DNA depletion and the other portion serving as the control without host DNA depletion. For the host depletion group, mammalian cells were lysed first to release host DNA which was then degraded by benzonase. Bacterial cells were lysed subsequently for the extraction of bacterial DNA and metagenomic sequencing. For the control group, total DNA was extracted after the lysis of host and bacterial cells.

**Figure 1:**
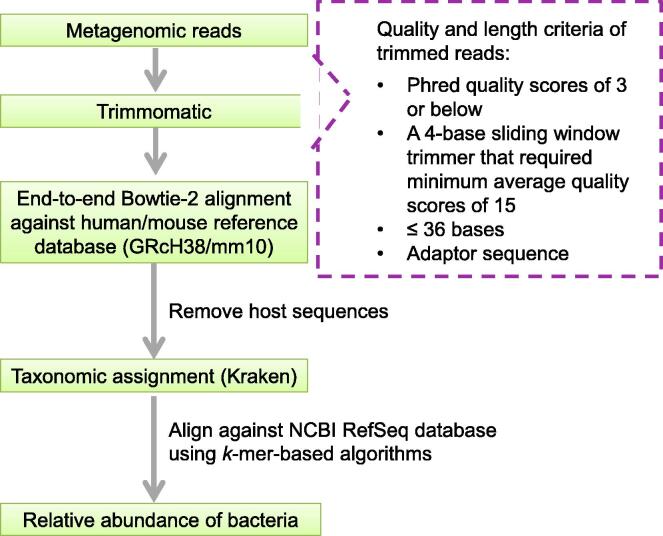
**Workflow of metagenomic analyses**

Bacterial DNA was extracted from colon biopsies after the removal of host DNA and an average of 55.37 ± 7.23 million reads were generated from 8 human (Table 1) and 19 mouse (Table S1) colon biopsy samples. The metagenomic data of human colon biopsies were aligned against a reference database of the human genome (GRcH38), whereas those of mouse tissues were aligned against a reference database of the mouse genome (mm10). After quality control and trimming of signals that were too low to have any biological significance, 95.02% ± 1.02% of the reads were shown to be derived from the human/mouse genome. With the removal of the host reads, the remaining reads were mapped to the National Center for Biotechnology Information (NCBI) standard set of microbial reference genomes for bacterial classification. The sequencing reads were aligned against the reference genome set using the Kraken taxonomic assignment software (Figure 1).

**Table 1:** **Alignment of metagenomic data collected from human colon biopsiesto reference genome database**

**Sample ID**	**Group**	**Input** **(No. of reads)**	**No. of reads mapped to host**	**Percentage of reads mapped to host**	**No. of reads mapped to bacterial genome**	**Percentage of reads mapped to bacterial genome**
N17	Non-depleted group	51,182,788	49,207,132	96.14%	732,578	1.43%
N2634		55,269,661	53,080,982	96.04%	781,754	1.41%
N2717		50,362,692	48,534,526	96.37%	657,313	1.31%
N4		55,828,293	53,829,640	96.42%	793,198	1.42%
P16		46,887,550	44,515,040	94.94%	828,820	1.77%
P17		56,477,519	54,512,101	96.52%	696,183	1.23%
P7		51,702,165	49,080,865	94.93%	844,197	1.63%
P9		46,482,759	42,647,931	91.75%	1,141,462	2.46%

N17	Depleted group	57,719,878	52,334,613	90.67%	1,727,554	2.99%
N2634		53,158,514	47,226,024	88.84%	2,081,664	3.92%
N2717		51,463,231	44,865,645	87.18%	2,120,978	4.12%
N4		47,535,052	42,662,709	89.75%	1,762,743	3.71%
P16		49,370,201	40,829,156	82.70%	2,669,819	5.41%
P17		55,056,508	52,022,894	94.49%	1,367,736	2.48%
P7		67,312,893	59,625,761	88.58%	2,716,743	4.04%
P9		49,879,677	43,150,909	86.51%	1,872,640	3.75%

*Note*: N, normal colon biopsy; P, polyp.

**Figure 2:**
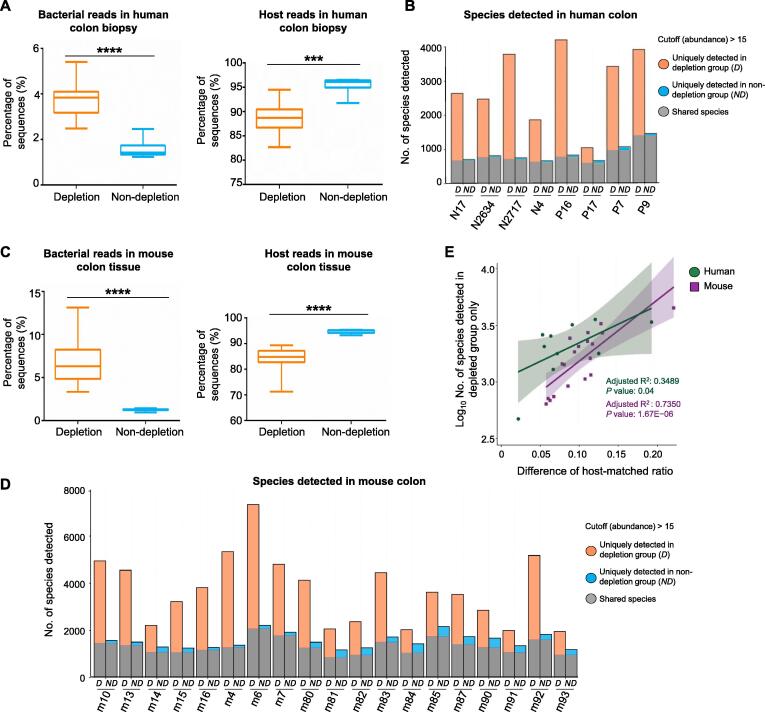
**Host DNA depletion enhanced the detection of bacterial species in colon tissues** **A.** The percentage of sequences mapped to the bacterial genome was increased while the percentage of sequences mapped to the human reference genome (GRcH38) was decreased after host DNA depletion in human colon biopsies (*N* = 8). **B.** More bacterial species were detected after removing host DNA from human colon biopsy. An average of 2998 species were detected in the depleted group whereas 891 species were detected in the non-depleted group. Specimen names with the prefix “N” and “P” represent normal biopsies and polyp biopsies, respectively. Each specimen was divided into host DNA depletion (*D*, left) and host DNA non-depletion (*ND*, right) groups. The shared species between the depleted group and the non-depleted group is colored gray. **C.** Increased reads were mapped to the bacterial genome after host DNA depletion in the mouse colon tissues (*N* = 19). A reduced number of sequences was mapped to the mouse reference genome (mm10). **D.** Enhanced detection of bacterial species was evident in the mouse colonic tissues that underwent host DNA depletion. An average of 3707 species were detected in the depleted group whereas 1555 species were detected in the non-depleted group. Specimen names with the prefix “m” represent mouse colon tissues. Each specimen was divided into host DNA depletion (*D*, left) and host DNA non-depletion (*ND*, right) groups. The shared species between the depleted group and the non-depleted group is colored gray. **E.** Correlation analysis suggested that the magnitude of host DNA depletion was strongly correlated with species detection by metagenomics. An increased number of species became detectable as more host DNA was removed from the tissue prior to metagenomic sequencing.

Removal of the host DNA increased the number of bacterial reads by 2.46 ± 0.20 folds while reducing host reads by 6.80% ± 1.06% (Figure 2A). With the cutoff value (read count > 15) (Figure S1), 2998 ± 401 (2.40 times more) bacterial species per sample were detected after host DNA depletion, whereas only 891 ± 98 species per sample were detected in the group without host DNA depletion (Figure 2B). Thus, significantly more bacterial species were detected upon host DNA depletion (*P* < 0.001). Most (93.45% ± 0.89%) of the bacterial species detected in the non-depleted group were also present in the depleted group, suggesting that our method increases the sensitivity for species detection without undermining the microbial composition of the tissue sample. Additionally, the abundances of bacteria detected only in the host DNA-depleted group were significantly lower compared with the abundances of shared bacteria between the two groups (*P* < 0.05) (Tables S2 and S3; Figure S2). The enhanced detection of bacterial species was confirmed by the metagenomic sequencing data from mouse colon tissues. Host DNA depletion protocol increased bacterial reads by 5.46 ± 0.42 folds while decreasing host reads by 10.2% ± 0.83% in the mouse colon tissues (Figure 2C). A total of 3707 ± 1465 bacterial species per sample were detected after host DNA depletion, whereas only 1555 ± 314 species per sample were detected in the non-depleted group (Figure 2D). In line with the human data, significantly more bacterial species were detected in the mouse colon tissue upon host DNA depletion (*P* < 0.001). 83.34% ± 7.00% of the bacteria detected in the non-depleted group were also present in the depleted group. Furthermore, correlation analysis of human and mouse samples suggested that the magnitude of host DNA depletion was strongly correlated with species detection by metagenomics (*P* < 0.05) (Figure 2E).

### Host DNA depletion increased the diversity of bacteria in colon tissues

**Figure 3:**
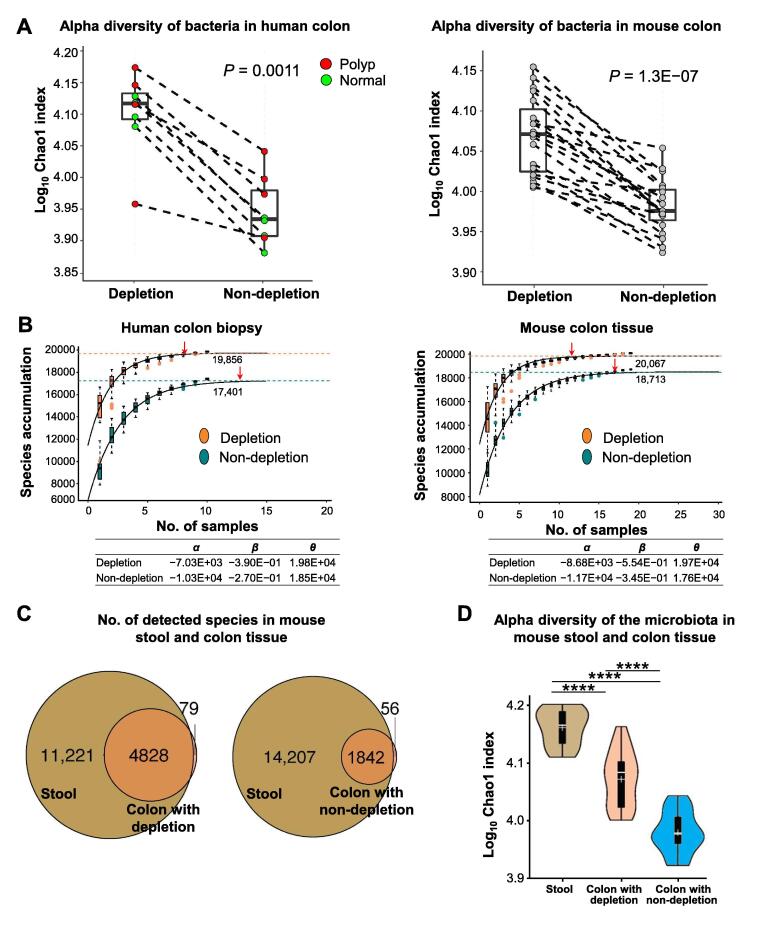
**Host DNA depletion increased the diversity of bacteria detected in colon tissue** **A.** Chao1 index was calculated to examine the bacterial diversity of human (*N* = 8) and mouse (*N* = 19) colon tissues before and after host DNA depletion. Increased alpha diversity was evident after host DNA depletion in human colon biopsies and in mouse colon tissues (*P* < 0.001). **B.** Species accumulation in the host DNA-depleted group reached the plateau sooner than the non-depleted group. Fitted curves were generated using a non-linear model $y=\alpha ∙e^{\beta x}+\theta$, where *x* is the number of samples and *y* is the number of detected species. The coefficients of the model were shown under the plot. The red arrow pointed to the saturated level. **C.** Majority of bacterial species identified in the mouse colon tissue (98.39% for the depleted group and 97.05% for the non-depleted group) were also found in the stool sample. **D.** The mouse stool microbiota exhibited the highest alpha diversity, followed by the host DNA-depleted gut microbiota and the non-depleted gut microbiota.

In concordance with improved species detection, an increased bacterial richness (measured by Chao1 index) was evident in the host DNA-depleted samples compared with non-depleted controls in human biopsies (*P* < 0.001) and mouse colon samples (*P* < 0.001) (Figure 3A and B). On the other hand, the beta diversity [measured by bray distance followed by principal coordinates analysis (PCoA)] revealed that the bacterial communities changed significantly upon host DNA removal, mainly because more bacteria were detected (Figure S3). Moreover, most of the bacterial species identified in the mouse colon tissue (98.39% for the depleted group and 97.05% for the non-depleted group) were also found in the stool sample (Figure 3C). The tissue-specific species [79 species in the depleted group (Table S4) and 56 species in the non-depleted group (Table S5)] belong to phyla Actinobacteria (36.71% in the depleted group and 46.43% in the non-depleted group) and Proteobacteria (37.97% in the depleted group and 32.14% in the non-depleted group). The detection of shared species in the colon and stool metagenomes suggested that no contamination was introduced into the samples during the host DNA depletion process. The stool microbiota exhibited the highest bacterial richness diversity, followed by the gut microbiota that had undergone host DNA depletion and the non-depleted gut microbiota (Figure 3D). The difference in bacterial richness among the metagenomes of stool, colon with host DNA depletion, and colon without host DNA depletion further supported that the amount of host DNA in the sample influenced the output of shotgun metagenomic sequencing and subsequent detection of bacterial species.

### Host DNA depletion increased the coverage of bacterial genes

**Figure 4:**
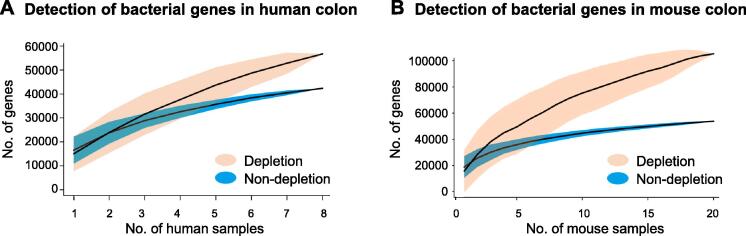
**Host DNA depletion increased the coverage of bacterial genes** **A.** Gene accumulation plot for the depleted group and the non-depleted group from human colon biopsies over different sample sizes. Upon host DNA depletion, detection of bacterial genes was increased by 33.89%. **B.** Gene accumulation plot for the depleted group and the non-depleted group from mouse colon tissues over different sample sizes. Detection of bacterial genes was increased by 95.75% after host DNA depletion.

To further validate the impact of removing host DNA prior to metagenomic sequencing on microbial analysis, we examined the bacterial genes detected in individual samples. Gene accumulation analysis demonstrated that the detection of bacterial genes was increased by 33.89% upon host DNA depletion in human colon biopsies (Figure 4A). This observation was confirmed by the data collected from mouse colon tissues. Detection of bacterial genes was increased by 95.75% in the depleted group compared with the non-depleted group (Figure 4B).

### Host DNA depletion maintained gut microbial relative abundance while increasing the sequencing depth of bacterial DNA

**Figure 5:**
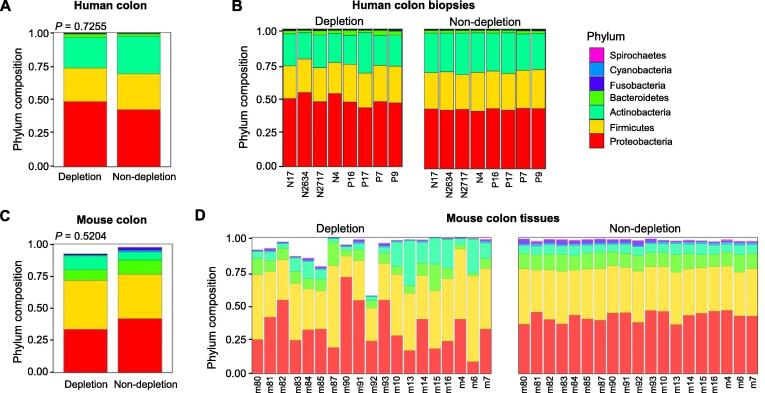
**The microbial community structure was maintained after host DNA depletion** **A.** Dominant phyla in the human colon biopsy. There was no significant difference in the dominant phyla between the depleted group and the non-depleted group. The *P* value was calculated by Fisher’s exact test with Monte Carlo simulation. **B.** Dominant phyla in individual human colon samples before and after host DNA depletion. **C.** Dominant phyla in the mouse colon tissue. The dominant phyla remained the same for the depleted group and the non-depleted group, suggesting that the host DNA depletion protocol does not alter the overall structure of microbial communities. The *P* value was calculated by Fisher’s exact test with Monte Carlo simulation. **D.** Dominant phyla in individual mouse colon samples before and after host DNA depletion.

**Figure 6:**
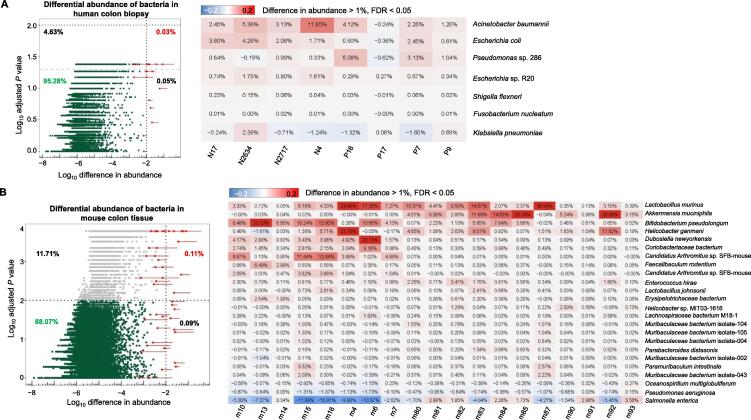
**Abundance shifts upon host DNA depletion** **A.** Abundance shifts were observed in several species after host DNA depletion in human colon biopsy. However, the bacteria showing abundance shifts upon host DNA depletion constituted only 0.03% of the entire microbial community, suggesting that the induced shifts have minimal impact on the relative abundance of the microbes. **B.** Abundance shifts were evident after removing host DNA from mouse colon tissue. The bacteria showing abundance shifts represented only 0.11% of the entire microbial community. FDR, false discovery rate.

The most abundant phyla in human colon biopsies from both groups included Proteobacteria, Firmicutes, Bacteroidetes, Actinobacteria, and Cyanobacteria (Figure 5A and B). There was no significant difference in the dominant phyla between the depleted group and the non-depleted group (*P* > 0.05, Fisher’s exact test), suggesting that the host DNA depletion did not alter the overall structure of microbial communities. The dominant phyla also remained unchanged after host DNA depletion in the mouse colon tissues (Figure 5C and D). At the species level, we observed that most of the bacteria (99.97% in human biopsies and 99.89% in mouse tissues) did not show significant differences (Figure 6). However, abundance shifts were observed in several species (0.03%) after removing host DNA. An enrichment of several Gram-negative bacteria, including *Acinetobacter baumannii*, *Escherichia coli*, and *Pseudomonas* sp. 286, was observed in human samples after removing host DNA using our protocol (Figure 6A). Likewise, the abundances of some bacterial species (< 0.11%) were altered in mouse samples undergoing host DNA depletion. Several species, such as *Lactobacillus murinus*, *Akkermansia muciniphila*, and *Bifidobacterium pseudolongum*, were enriched whereas other species, such as *Salmonella enterica* and *E. coli*, were depleted after removing host DNA from the mouse colon tissues (Figure 6B). Given that the bacteria showing abundance shifts upon host DNA depletion constituted only 0.03% of the entire microbial communities in human samples and 0.11% in mouse samples, the induced abundance shifts had a minimal impact on the structure of the microbial communities. Furthermore, the quantitative polymerase chain reaction (qPCR) result showed that our method depleted host DNA and extracted bacterial DNA as effectively as other methods using osmotic lysis or saponin treatment (Figure S4). We calculated the reduced ratio, referring to the difference between the host DNA ratio in the depleted group and in the non-depleted group. We observed the reduced ratio from the qPCR data (9.46%) was similar to that from metagenomic sequencing data (10%). These findings suggested that our method improved the sequencing depth of bacterial DNA, uncovering low-abundance bacterial species that may have significant biological functions in health maintenance or disease development.

## Discussion

Overwhelming quantities of host DNA relative to microbial DNA hinder the detection of specific bacteria in tissue samples by shotgun metagenomic sequencing. As such, we explored the various steps of DNA extraction and described an optimized protocol for reducing host DNA in the colon tissue and enhancing the sequencing output of bacterial DNA. Previous studies have used AHL (QIAGEN) lysis buffer to differentially lyse host cells and degraded host DNA by benzonase prior to extracting bacterial DNA from saliva or sputum samples [8], [18], [19]. Unlike these studies using samples with high microbial biomass (proportion of microbial DNA in total DNA), we extracted DNA from samples with much lower biomass, such as the limited colon biopsies from human. We included an additional step to pre-treat the biopsy tissue with collagenase D and built our own DNA libraries for metagenomic sequencing using only 1 ng of input DNA. Our metagenomic data demonstrate that our method increases the sequencing depth of bacterial DNA, leading to enhanced detection of bacterial species in tissue samples. We improved an existing protocol for the extraction of bacterial DNA from the biopsy tissue. Our method will be a useful tool to process clinical samples, especially biopsy tissues which tend to be in limited amounts, to effectively deplete host DNA and enhance bacteria detection in the samples.

The novelty of our current study was that only a small quantity of the tissue was required for host DNA depletion and consequent shotgun metagenomic sequencing. In most cases, acquiring substantial tissue samples for microbiome research is challenging, particularly when using human tissue biopsies. In this regard, we performed the host DNA depletion on ∼ 2.5 mg of colon biopsy whereas Nelson et al. and Charalampous et al. performed host DNA depletion on 200 mg and 400 μl of sputum samples, respectively [8], [18]. We calculated the ratio of bacterial reads in total reads and found that it was comparable but somewhat improved than the ratio reported by Nelson et al., in which they found a 5-fold increase in bacterial reads and a 28% reduction in host reads upon host DNA depletion [8]. Likewise, our protocol increased bacterial reads by 5.5-fold and decreased host reads by 10% in the mouse colon tissue. We also reported a 2.5-fold increase in bacterial reads and a 6.8% decrease in host reads in human colon biopsy undergoing host DNA depletion. Our data also showed a small amount of microorganism DNA depletion along with the host DNA depletion, and this could be due to the disrupted microorganism upon freezing and thawing, or extracellular DNA from dead bacteria. However, the level of depleting microorganism DNA was far less than the host DNA depletion.

Another benefit of our method was that more tissue can be reserved for other analyses, including transcriptomics, proteomics, and metabolomics, to gain insights into the microbial function and activity [21], [22], [23]. Our method increased alpha diversity and detected new species absent from the non-depleted controls, while maintaining the overall structure of the microbial communities. There were no significant changes in the dominant phyla of the human and mouse colon undergoing our depletion protocol. Hence, our protocol maintained gut microbial composition while increasing the sequencing depth of bacterial DNA.

Recent studies have developed an alternative approach to differential lysis of host and bacterial cells via separating host DNA from bacterial DNA by methyl-CpG binding domain (MBD-Fc) paramagnetic beads [24]. Removing host DNA based on the abundance of CpG methylation was reported by Feehery et al. to evaluate the enriched bacterial DNA in human saliva and blood samples [24]. A later study has highlighted the limitation of the methylation-based approach, including bias toward the recovery of CpG-rich and long DNA templates [25]. In addition, Feehery et al. used a DNA extraction protocol devoid of mechanistic disruption of the cell wall. The absence of the bead-beating process in their protocol hinders the detection of Gram-positive bacteria with the thick cell wall. As such, our method comprised enzymatic and mechanistic lysis of bacterial cells and extracted bacterial DNA more efficiently.

A bacterial mock community has been recently constructed to examine the changes of specific taxa upon host DNA depletion [8], [18]. In contrast to these studies that focused on the impact of host DNA depletion on selective pathogens, our work provided an unbiased examination and comparison of the microbial profile using limited tissue biopsies. Moreover, our depletion method reduced host DNA and enhanced microbial composition of colon tissues, supporting its utility in future relevant research with more accurate characterization of the colon tissue metagenome.

In conclusion, we described an improved method that removes host DNA from colon tissue and enhances the sensitivity of shotgun metagenomic sequencing in bacteria detection in the biopsy tissue. Using this method to remove host DNA from clinical samples may yield a more accurate taxonomic profile of the gut microbiota, leading to the identification of bacteria that are important for disease initiation or progression.

## Materials and methods

### Sample collection

Colon biopsies (2–3 biopsies per case) were collected from eight human participants during colonoscopy at the Prince of Wales Hospital of the Chinese University of Hong Kong and stored at −80 °C until DNA extraction. Colon tissues were also collected in 19 C57BL/6 mice at the age of 6–8 weeks and stored at −80 °C until DNA extraction.

### Host DNA depletion and bacterial DNA extraction

Tissue biopsies were homogenized and divided into two portions equally, with one portion undergoing host DNA depletion and the other portion serving as the control without host DNA depletion. For the group with host DNA depletion, host DNA was removed through differential lysis of mammalian and bacterial cells prior to sequencing. The mammalian cells were lysed in the initial lysis steps, whereas the bacterial cells were protected from the initial lysis by their thick cell wall. Host DNA was then degraded by benzonase, and bacterial DNA was isolated through a combination of enzymatic lysis, bead-beating procedure, and column-based purification. In brief, ∼ 2.5-mg colon tissues were pre-treated with collagenase D (2 mg/ml) at 37 °C for 1 h followed by DNA extraction using the QIAamp DNA microbiome kit (Catalog No. 51704, QIAGEN). 500 μl of AHL buffer was added to the sample and incubated for 30 min at room temperature with end-over-end rotation. The sample was centrifuged at 10,000 *g* for 10 min and the supernatant was removed. 190 μl of RDD (QIAGEN) buffer and 2.5 μl of benzonase were added to the sample and incubated at 37 °C for 30 min. The sample was then incubated with 20 μl proteinase K at 56 °C for 30 min. 200 μl ATL (QIAGEN) buffer (containing reagent DX) was added to the sample, and the whole mixture was transferred to pathogen lysis tube L. Sample was lysed mechanically by Bioprep-24 homogenizer (Hangzhou Allsheng Instruments, Hangzhou, China) with a velocity of 6.5 m/s three times for 45 s with a 5-min intermission, while the sample was stored on ice. The supernatant was transferred to a fresh tube, and 40 μl proteinase K was added and incubated at 56 °C for 30 min. The sample was incubated with 200 μl of APL2 (QIAGEN) buffer at 70 °C for 10 min. 200 μl ethanol was added to the sample, and the whole mixture was transferred to the QIAamp UCP mini spin column. The column was centrifuged at 6000 *g* for 1 min and then washed with AW1 and AW2 (QIAGEN) buffer. DNA was eluted in 30 μl water.

For the control group without host DNA depletion, total DNA was extracted from ∼ 2.5 mg of colon tissue by a combination of enzymatic and mechanical lysis of host and bacterial cells. Tissues were treated with lysozyme (10 mg/ml) at 37 °C for 1 h, followed by mechanical lysis using 100-mg glass beads (Catalog No. G4649, Sigma-Aldrich) and Bioprep-24 homogenizer (Hangzhou Allsheng Instruments) at a velocity of 6.5 m/s three times for 45 s with a 5-min intermission. Total DNA was then isolated using the QIAamp DNA mini Kit (Catalog No. 51304, QIAGEN). In brief, 100 μl ATL buffer and 20 μl proteinase K were added to the sample and incubated at 56 °C for 2 h. 4 μl RNaseA was added to the sample and incubated at room temperature for 2 min. 200 μl AL buffer was added to the sample and incubated at 70 °C for 10 min. After the addition of 200 μl ethanol, the whole mixture was transferred to the QIAamp mini spin column and centrifuged at 6000 *g* for 1 min. The sample was then washed with AW1 and AW2 buffer, and eluted in 100 μl water. After DNA extraction, DNA concentration was measured by Quantus fluorometer (Catalog No. E6150, Promega). The step-by-step host DNA depletion protocol is provided in File S1.

### Library preparation and metagenomic sequencing

DNA libraries were prepared and subjected to shotgun metagenomic sequencing for taxonomic profiling using the NEBNext Ultra II FS DNA library prep kit for Illumina (Catalog No. E6177, New England Biolabs). 1 ng of DNA was fragmented with fragmentation enzyme for 10 min at 37 °C followed by adaptor ligation. Adaptor was diluted 25-fold, and 2.5 μl adaptor was incubated with 35 μl fragmented DNA, 30 μl NEBNext Ultra II ligation master mix, and 1 μl NEBNext ligation enhancer at 20 °C for 15 min. 3 μl of USER enzyme was added to the ligation mixture and incubated at 37 °C for 15 min. Purification with beads was carried out according to the manufacturer’s instructions prior to PCR enrichment of adaptor-ligated DNA under the following conditions: an initial temperature of 98 °C for 30 s followed by 12 cycles of 98 °C for 10 s and 65 °C for 75 s, then 65 °C for 5 min. Shotgun metagenomic sequencing was performed at Novogene Technology Beijing by Illumina HiSeq 2000 platform (Illumina) with paired-end 150 bp (PE150).

### Metagenomic sequence analysis and taxonomic assignment

Metagenomic reads were quality-filtered using Trimmomatic, version 0.36 [26]. Any leading or trailing N-bases and other bases that had Phred quality scores of 3 or below, sequence reads with a 4-base sliding window trimmer that required minimum average quality scores of 15, sequence reads that had 36 bases or fewer, and fragments of adapter sequences were trimmed. To remove the host sequences, we performed end-to-end Bowtie-2 [27] alignment with “very-sensitive” preset options against a reference database of the human genome (GRcH38) or mouse genome (mm10). The remaining non-host reads were aligned to the reference database of the bacterial genome from the NCBI RefSeq database. We assigned metagenomic reads to microbial taxa using the *k*-mer-based algorithms as implemented in the Kraken [28] taxonomic annotation pipeline. Microbial richness was measured by the Chao1 index. Beta diversity was accessed by Bray-Curtis distance, and the PCoA was used for ordination analysis.

### Quantification of host DNA and bacterial DNA in tissue samples by qPCR

qPCR was performed to evaluate the level of host DNA and bacterial DNA in mouse colon tissues treated with different depletion methods. 1 μl genomic DNA was mixed with 10 µl universal SYBR green PCR master mix (Catalog No. RR420A, TaKaRa) on the QuantStudio 7 Flex system (Thermo Fisher Scientific). The amount of DNA in the sample was calculated using standard curves. Nucleotide sequences of primers were: 16S (forward primer: 5′-GCAGGCCTAACACATGCAAGTC-3′; reverse primer: 5′-CTGCTGCCTCCCGTAGGAGT-3′) and mouse *β*-actin (forward primer: 5′-CATTGCTGACAGGATGCAGAAGG-3′; reverse primer: 5′-TGCTGGAAGGTGGACAGTGAGG-3′).

### Calculation of reduced host DNA ratio upon host DNA depletion

Let *x* and *y* represent the input DNA content (ng) for host and microorganisms, respectively. We speculated that DNA-reduced factors are $k_{\mathrm{host}}$ for host and $k_{\mathrm{micro}}$ for microorganisms after host DNA depletion, with $c=\frac{k_{\mathrm{host}}}{k_{\mathrm{micro}}}$, where *c* is a constant, associated with the treatment (*e.g.*, protocol and treatment time). From the qPCR data (the reduced factor for host DNA by around 10,000 and for bacterial DNA by around 50), we let $k_{\mathrm{host}}=200t$ and $k_{\mathrm{micro}}=t$ (*c* = 200). Then, the host ratio without host DNA depletion is $\frac{x}{x+y}$, whereas the host ratio with host DNA depletion is $\frac{x/200t}{x/200t+y/t}$. The difference between the two host ratios is $\frac{199xy}{\left(x+y\right)\left(x+200y\right)}$, indicating that reduced host DNA ratio is determined by the input DNA content and the constant *c*.

### Statistical analysis

The numerical variables between the two groups were compared using two-tailed Student’s *t*-test or Mann-Whitney *U* test where appropriate. Repeated measurement data were analyzed by two-way ANOVA test. All statistical analyses were conducted using computing R (version 3.6.3). Differences were considered significant if *P* values were less than 0.05.

## Ethical statement

Written consents were obtained from all human participants. The study was approved by the Clinical Research Ethics Committee of the Chinese University of Hong Kong, China (Approval No. 2018.359). The animal experiments were approved by the Animal Experimentation Ethics Committee of the Chinese University of Hong Kong, China (Approval No. 19-054-MIS-4-B).

## Data availability

The datasets generated in the current study are available in the Genome Sequence Archive [29] at the National Genomics Data Center, Beijing Institute of Genomics, Chinese Academy of Sciences / China National Center for Bioinformation (GSA: CRA005229; GSA-Human: HRA001685), and are publicly accessible at https://ngdc.cncb.ac.cn/gsa and https://ngdc.cncb.ac.cn/gsa-human, respectively.

## Competing interests

The authors declare no competing interests.

## CRediT authorship contribution statement

**Wing Yin Cheng:** Methodology, Formal analysis, Investigation, Writing – original draft, Visualization. **Wei-Xin Liu:** Data curation, Formal analysis, Investigation, Writing – original draft, Visualization. **Yanqiang Ding:** Formal analysis, Investigation. **Guoping Wang:** Validation, Investigation, Writing – review & editing. **Yu Shi:** Validation, Investigation, Writing – review & editing. **Eagle S.H. Chu:** Resources. **Sunny Wong:** Resources. **Joseph J.Y. Sung:** Writing – review & editing. **Jun Yu:** Conceptualization, Writing – review & editing, Funding acquisition. All authors have read and approved the final manuscript.

## Supplementary material

Supplementary File S1 **Step-by-step protocol**

Supplementary Figure S1 **The detected species at different cutoff A.** Ratio of bacteria species detected only in the depleted group in all bacteria at different cutoffs. **B.** Ratio of shared bacteria (between the depleted group and non-depleted group) in the non-depleted group at different cutoffs.

Supplementary Figure S2 **The bacteria detected only in the depleted group** Comparison of relative abundance between the shared bacteria (between the depleted group and non-depleted group) and the bacteria detected only in the depleted group (unique).

Supplementary Figure S3 **PCoA plot for samples from human colon biopsy (left) and mouse colon tissue (right) upon host DNA depletion**

Supplementary Figure S4 **Comparison of methods using different cell lysis A.** We applied different methods to mouse colon tissues (N = 5). Our method, which uses AHL buffer to lyse host cells prior to extraction of bacterial DNA, depleted host DNA as effectively as other methods using osmotic lysis or saponin treatment. **B.** Our method extracted a similar amount of bacterial DNA as the other depletion methods. **C.** The ratio of host DNA to bacterial DNA obtained using our method was similar to that obtained by the other depletion methods.

Supplementary Table S1 **Alignment of metagenomic data collected from mouse colon biopsies onto reference genome database**

Supplementary Table S2 **Microbes that are only identified in host DNA-depleted samples compared with non-depleted samples (union set among all specimens)**

Supplementary Table S3 **Microbes that are only identified in non-depleted samples compared with host DNA-depleted samples (union set among all specimens)**

Supplementary Table S4 **Taxonomic ranks for the tissue-specific species in the depleted group**

Supplementary Table S5 **Taxonomic ranks for the tissue-specific species in the non-depleted group**

## References

[1] Human Microbiome Project Consortium (2012). Structure, function and diversity of the healthy human microbiome. Nature.

[2] Dilthey A.T., Jain C., Koren S., Phillippy A.M. (2019). Strain-level metagenomic assignment and compositional estimation for long reads with MetaMaps. Nat Commun.

[3] Goltsman D.S.A., Sun C.L., Proctor D.M., DiGiulio D.B., Robaczewska A., Thomas B.C. (2018). Metagenomic analysis with strain-level resolution reveals fine-scale variation in the human pregnancy microbiome. Genome Res.

[4] Truong D.T., Tett A., Pasolli E., Huttenhower C., Segata N. (2017). Microbial strain-level population structure and genetic diversity from metagenomes. Genome Res.

[5] Mac Aogain M., Narayana J.K., Tiew P.Y., Ali N., Yong V.F.L., Jaggi T.K. (2021). Integrative microbiomics in bronchiectasis exacerbations. Nat Med.

[6] Nakatsu G., Li X., Zhou H., Sheng J., Wong S.H., Wu W.K. (2015). Gut mucosal microbiome across stages of colorectal carcinogenesis. Nat Commun.

[7] Coker O.O., Dai Z., Nie Y., Zhao G., Cao L., Nakatsu G. (2018). Mucosal microbiome dysbiosis in gastric carcinogenesis. Gut.

[8] Nelson M.T., Pope C.E., Marsh R.L., Wolter D.J., Weiss E.J., Hager K.R. (2019). Human and extracellular DNA depletion for metagenomic analysis of complex clinical infection samples yields optimized viable microbiome profiles. Cell Rep.

[9] Chiu C.Y., Miller S.A. (2019). Clinical metagenomics. Nat Rev Genet.

[10] Gu W., Miller S., Chiu C.Y. (2019). Clinical metagenomic next-generation sequencing for pathogen detection. Annu Rev Pathol.

[11] Cummings L.A., Kurosawa K., Hoogestraat D.R., SenGupta D.J., Candra F., Doyle M. (2016). Clinical next generation sequencing outperforms standard microbiological culture for characterizing polymicrobial samples. Clin Chem.

[12] Zaheer R., Noyes N., Ortega Polo R., Cook S.R., Marinier E., Van Domselaar G. (2018). Impact of sequencing depth on the characterization of the microbiome and resistome. Sci Rep.

[13] Naccache S.N., Peggs K.S., Mattes F.M., Phadke R., Garson J.A., Grant P. (2015). Diagnosis of neuroinvasive astrovirus infection in an immunocompromised adult with encephalitis by unbiased next-generation sequencing. Clin Infect Dis.

[14] Couto N., Schuele L., Raangs E.C., Machado M.P., Mendes C.I., Jesus T.F. (2018). Critical steps in clinical shotgun metagenomics for the concomitant detection and typing of microbial pathogens. Sci Rep.

[15] Hasan M.R., Rawat A., Tang P., Jithesh P.V., Thomas E., Tan R. (2016). Depletion of human DNA in spiked clinical specimens for improvement of sensitivity of pathogen detection by next-generation sequencing. J Clin Microbiol.

[16] Gu W., Crawford E.D., O'Donovan B.D., Wilson M.R., Chow E.D., Retallack H. (2016). Depletion of abundant sequences by hybridization (DASH): using Cas9 to remove unwanted high-abundance species in sequencing libraries and molecular counting applications. Genome Biol.

[17] Thoendel M., Jeraldo P.R., Greenwood-Quaintance K.E., Yao J.Z., Chia N., Hanssen A.D. (2016). Comparison of microbial DNA enrichment tools for metagenomic whole genome sequencing. J Microbiol Methods.

[18] Charalampous T., Kay G.L., Richardson H., Aydin A., Baldan R., Jeanes C. (2019). Nanopore metagenomics enables rapid clinical diagnosis of bacterial lower respiratory infection. Nat Biotechnol.

[19] Marotz C.A., Sanders J.G., Zuniga C., Zaramela L.S., Knight R., Zengler K. (2018). Improving saliva shotgun metagenomics by chemical host DNA depletion. Microbiome.

[20] Heravi F.S., Zakrzewski M., Vickery K., Hu H. (2020). Host DNA depletion efficiency of microbiome DNA enrichment methods in infected tissue samples. J Microbiol Methods.

[21] Lloyd-Price J., Arze C., Ananthakrishnan A.N., Schirmer M., Avila-Pacheco J., Poon T.W. (2019). Multi-omics of the gut microbial ecosystem in inflammatory bowel diseases. Nature.

[22] Zhou W., Sailani M.R., Contrepois K., Zhou Y., Ahadi S., Leopold S.R. (2019). Longitudinal multi-omics of host-microbe dynamics in prediabetes. Nature.

[23] Heintz-Buschart A., May P., Laczny C.C., Lebrun L.A., Bellora C., Krishna A. (2016). Integrated multi-omics of the human gut microbiome in a case study of familial type 1 diabetes. Nat Microbiol.

[24] Feehery G.R., Yigit E., Oyola S.O., Langhorst B.W., Schmidt V.T., Stewart F.J. (2013). A method for selectively enriching microbial DNA from contaminating vertebrate host DNA. PLoS One.

[25] Seguin-Orlando A., Gamba C., Sarkissian C., Ermini L., Louvel G., Boulygina E. (2015). Pros and cons of methylation-based enrichment methods for ancient DNA. Sci Rep.

[26] Bolger A.M., Lohse M., Usadel B. (2014). Trimmomatic: a flexible trimmer for Illumina sequence data. Bioinformatics.

[27] Langmead B., Salzberg S.L. (2012). Fast gapped-read alignment with Bowtie 2. Nat Methods.

[28] Wood D.E., Lu J., Langmead B. (2019). Improved metagenomic analysis with Kraken 2. Genome Biol.

[29] Chen T., Chen X., Zhang S., Zhu J., Tang B., Wang A. (2021). Genome Sequence Archive Family: toward explosive data growth and diverse data types. Genomics Proteomics Bioinformatics.

