# Supplementary material for: High Sensitivity of Shotgun Metagenomic Sequencing in Colon Tissue Biopsy by Host DNA Depletion
Source: Genomics Proteomics Bioinformatics. 2022 Sep 26;21(6):1195–205. doi: 10.1016/j.gpb.2022.09.003 (PMC11082407; doi:10.1016/j.gpb.2022.09.003)
Supplement: Supplementary File S1 — Step-by-step protocol [file mmc1.docx]

**File S1 Host DNA depletion protocol**

**Reagents**

| **Reagent** | **Manufactuer** | **Catalog ID** |
| --- | --- | --- |
| Collagenase D | Sigma-Aldrich | COLLD-RO |
| QIAamp DNA microbiome kit | QIAGEN | 51704 |
| NEBNext ultra II FS DNA library prep kit for Illumina | New England Biolabs | E6177 |

**Protocol**

**Depletion of host DNA and isolation of bacterial DNA**

1. Prepare 2 mg/mL of collagenase D using PBS. Filter the solution with 0.22 uM filter.
2. Pre-treat ~ 2.5 mg colon tissues with 1 mL of collagenase D (2 mg/mL) at 37 ˚C for one hour.
3. Add 500 uL AHL buffer to the sample and incubate 30 min at room temperature with end-over-end rotation.
4. Centrifuge the sample at 10,000 × g for 10 min and remove the supernatant.
5. 190 uL of RDD buffer and 2.5 uL of benzonase were added to sample and incubated at 37 ˚C for 30 min.
6. Add 20 uL Proteinase K to sample and incubate at 56 ˚C for 30 min.
7. Add 200 uL ATL buffer (containing reagent DX) to sample and transfer the whole mixture to pathogen lysis tube L.
8. Sample was lysed mechanically by Bioprep-24 homogenizer (Allsheng Instruments) with a velocity of 6.5 m/s three times for 45 s with a 5-min intermission, while sample was stored on ice. Supernatant was transferred to a fresh tube and 40 uL proteinase K was added and incubated at 56 ˚C for 30 min.
9. Incubate sample with 200 uL of APL2 buffer at 70 ˚C for 10 min.
10. Add 200 uL ethanol to sample and transfer the whole mixture to the QIAamp UCP Mini spin column.
11. Centrifuge the column at 6000 × g for 1 min and wash with AW1 and AW2 buffer.
12. Elute DNA in 30 uL water.
13. Measure DNA concentration by Quantus fluorometer (Promega, Cat. No. E6150).

**Library preparation for metagenomic sequencing**

1. 1 ng of DNA was fragmented with fragmentation enzyme for 10 min at 37 ˚C.
2. Adaptor was diluted by 25-fold and 2.5 uL adaptor was incubated with 35 uL fragmented DNA, 30 uL NEBNext ultra II ligation master mix, and 1 uL NEBNext ligation enhancer at 20 ˚C for 15 min.
3. Add 3 uL of USER enzyme to the ligation mixture and incubate at 37 ˚C for 15 min.
4. Purification with beads was carried out according to manufacturer’s instructions prior to PCR enrichment of adaptor-ligated DNA under the following conditions: an initial temperature 98 ˚C for 30 s followed by 12 cycles of 98 ˚C for 10 s and 65 ˚C for 75 s, then 65 ˚C for 5 min.
5. Shotgun metagenomic sequencing was performed at Novogene Technology Beijing by Illumina HiSeq 2000 platform (Illumina) with paired-end 150 bp (PE150).
