# Supplementary material for: High Sensitivity of Shotgun Metagenomic Sequencing in Colon Tissue Biopsy by Host DNA Depletion
Source: Genomics Proteomics Bioinformatics. 2022 Sep 26;21(6):1195–205. doi: 10.1016/j.gpb.2022.09.003 (PMC11082407; doi:10.1016/j.gpb.2022.09.003)
Supplement: Supplementary Figure S1 — The detected species at different cutoff A. Ratio of bacteria species detected only in the depleted group in all bacteria at different cutoffs. B. Ratio of shared bacteria (between the depleted group and non-depleted group) in the non-depleted group at different cutoffs. [file mmc2.pptx]

## Slide 1
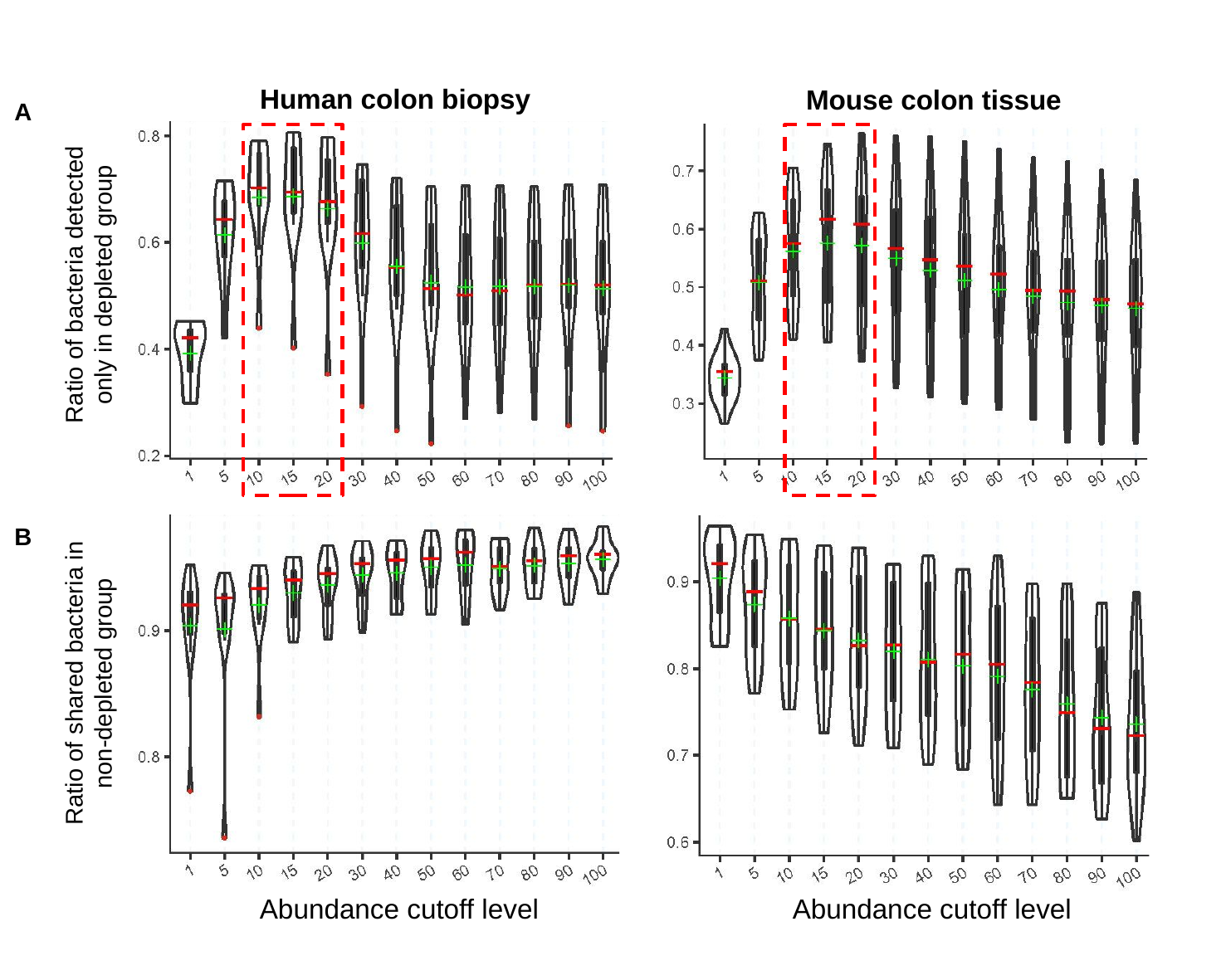

Human colon biopsy
Mouse colon tissue
A
Ratio of bacteria detected only in depleted group
B
Ratio of shared bacteria in non-depleted group
Abundance cutoff level
Abundance cutoff level
