# Supplementary material for: High Sensitivity of Shotgun Metagenomic Sequencing in Colon Tissue Biopsy by Host DNA Depletion
Source: Genomics Proteomics Bioinformatics. 2022 Sep 26;21(6):1195–205. doi: 10.1016/j.gpb.2022.09.003 (PMC11082407; doi:10.1016/j.gpb.2022.09.003)
Supplement: Supplementary Figure S2 — The bacteria detected only in the depleted group Comparison of relative abundance between the shared bacteria (between the depleted group and non-depleted group) and the bacteria detected only in the depleted group (unique). [file mmc3.pptx]

## Slide 1
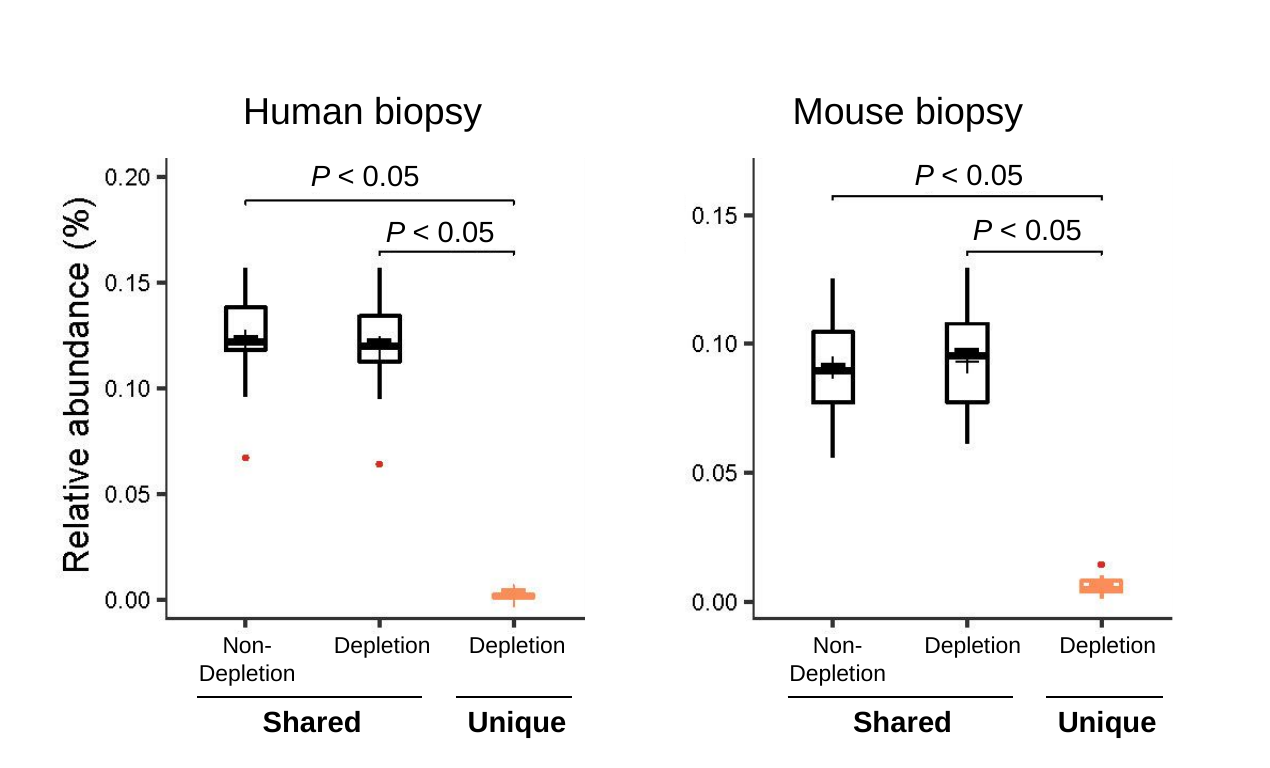

Human biopsy
Mouse biopsy
P < 0.05
P < 0.05
Non-
Depletion
Depletion
Depletion
Non-
Depletion
Depletion
Depletion
Shared
Unique
Shared
Unique
P < 0.05
P < 0.05
