# Supplementary figures and images for: High Sensitivity of Shotgun Metagenomic Sequencing in Colon Tissue Biopsy by Host DNA Depletion
Source: Genomics Proteomics Bioinformatics. 2022 Sep 26;21(6):1195–205. doi: 10.1016/j.gpb.2022.09.003 (PMC11082407; doi:10.1016/j.gpb.2022.09.003)

## Slide 1
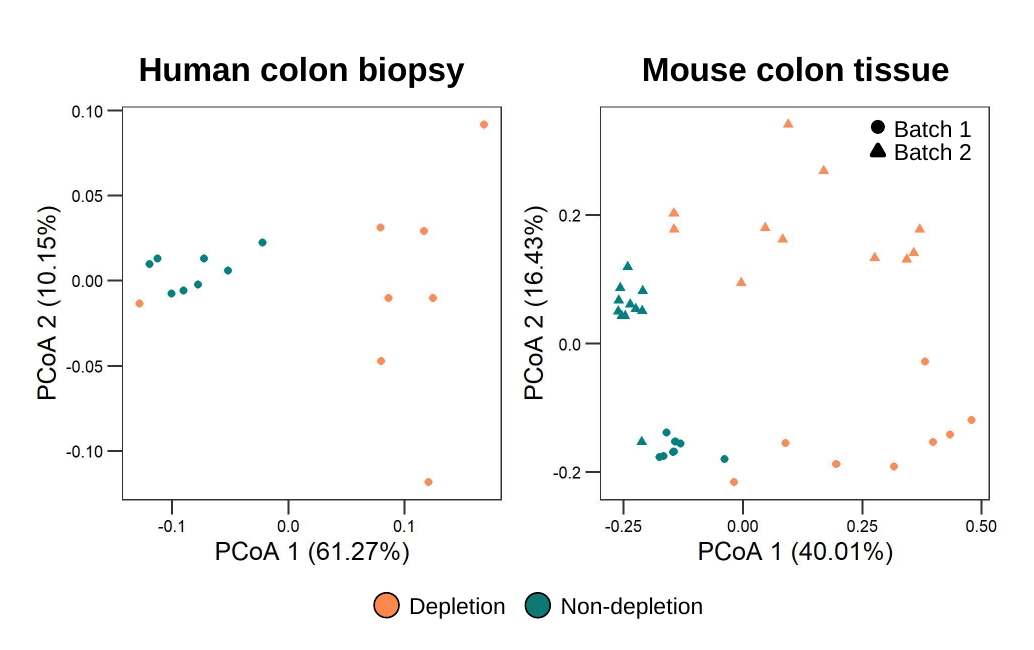

Human colon biopsy
Mouse colon tissue
Batch 1
Batch 2
Depletion
Non-depletion

Supplement: Supplementary Figure S3 — PCoA plot for samples from human colon biopsy (left) and mouse colon tissue (right) upon host DNA depletion [file mmc4.pptx]
