# Supplementary material for: High Sensitivity of Shotgun Metagenomic Sequencing in Colon Tissue Biopsy by Host DNA Depletion
Source: Genomics Proteomics Bioinformatics. 2022 Sep 26;21(6):1195–205. doi: 10.1016/j.gpb.2022.09.003 (PMC11082407; doi:10.1016/j.gpb.2022.09.003)
Supplement: Supplementary Figure S4 — Comparison of methods using different cell lysis A. We applied different methods to mouse colon tissues (N = 5). Our method, which uses AHL buffer to lyse host cells prior to extraction of bacterial DNA, depleted host DNA as effectively as other methods using osmotic lysis or saponin treatment. B. Our method extracted a similar amount of bacterial DNA as the other depletion methods. C. The ratio of host DNA to bacterial DNA obtained using our method was similar to that obtained by the other depletion methods. [file mmc5.pptx]

## Slide 1
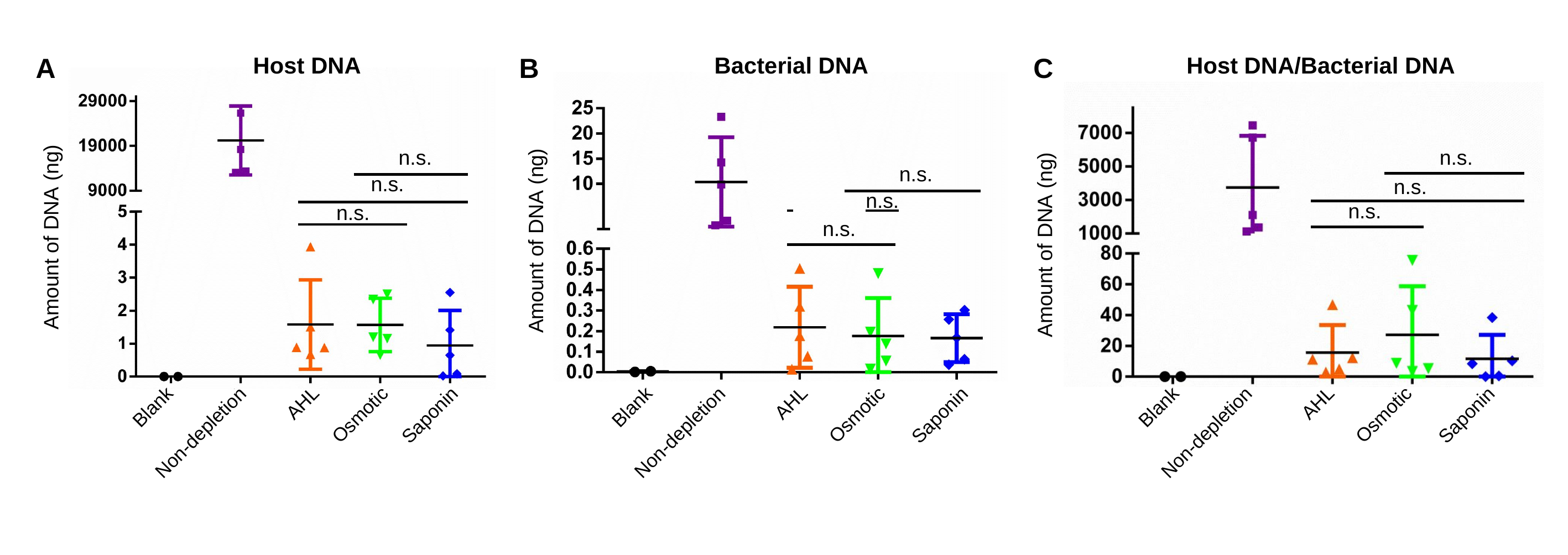

A
Host DNA
B
Bacterial DNA
C
Host DNA/Bacterial DNA
n.s.
n.s.
n.s.
Amount of DNA (ng)
Amount of DNA (ng)
Amount of DNA (ng)
n.s.
n.s.
 	n.s.
n.s.
n.s.
n.s.
AHL
AHL
AHL
Blank
Blank
Blank
Osmotic
Osmotic
Osmotic
Saponin
Saponin
Saponin
Non-depletion
Non-depletion
Non-depletion
